# Early Switch From Intravenous to Oral Antibiotics for Patients With Uncomplicated Gram-Negative Bacteremia

**DOI:** 10.1001/jamanetworkopen.2023.52314

**Published:** 2024-01-23

**Authors:** Sandra Tingsgård, Simone Bastrup Israelsen, Henrik Løvendahl Jørgensen, Christian Østergaard, Thomas Benfield

**Affiliations:** 1 Center of Research & Disruption of Infectious Diseases, Department of Infectious Diseases, Copenhagen University Hospital–Amager and Hvidovre, Hvidovre, Denmark; 2 Department of Clinical Biochemistry, Copenhagen University Hospital–Amager and Hvidovre, Hvidovre, Denmark; 3 Department of Clinical Medicine, University of Copenhagen, Copenhagen, Denmark; 4 Department of Clinical Microbiology, Copenhagen University Hospital–Amager and Hvidovre, Hvidovre, Denmark

## Abstract

**Importance:**

Gram-negative bacteremia is a global health concern, and optimizing the transition from intravenous (IV) to oral antibiotics is a critical step in improving patient treatment and resource utilization.

**Objective:**

To assess the association of switching to oral antibiotics within 4 days after initial blood culture with 90-day all-cause mortality compared with prolonged IV antibiotic treatment for patients with uncomplicated gram-negative bacteremia.

**Design, Setting, and Participants:**

This cohort study conducted using the target trial emulation framework included observational data from adults with uncomplicated gram-negative bacteremia in 4 hospitals in Copenhagen, Denmark, from January 1, 2018, through December 31, 2021. The duration of follow-up was 90 days. Eligibility criteria included a blood culture positive for growth of gram-negative bacteria, clinical stability within 4 days of initial blood culture, an available susceptibility report on day 4, and initiation of appropriate empirical IV antibiotic treatment within 24 hours of blood culture.

**Exposure:**

Switching to oral antibiotics within 4 days after initial blood culture compared with continuing IV antibiotic treatment for at least 5 days after initial blood culture.

**Main Outcomes and Measures:**

The main outcome was 90-day all-cause mortality. Inverse probability of treatment weighting was applied to adjust for confounding. Intention-to-treat and per-protocol analyses were performed using pooled logistic regression to estimate absolute risk, risk difference (RD), and risk ratio (RR); 95% CIs were computed using bootstrapping.

**Results:**

A total of 914 individuals were included in the target trial emulation analysis (512 [56.0%] male; median age, 74.5 years [IQR, 63.3-83.2 years]); 433 (47.4%) transitioned early to oral antibiotic treatment, and 481 (52.6%) received prolonged IV treatment. Ninety-nine individuals (10.8%) died during follow-up. The proportion of individuals who died was higher in the group receiving prolonged IV treatment (69 [14.3%] vs 30 [6.9%]). In the intention-to-treat analysis, 90-day all-cause mortality risk was 9.1% (95% CI, 6.7%-11.6%) for the early-switch group and 11.7% (95% CI, 9.6%-13.8%) for the group receiving prolonged IV treatment; the RD was −2.5% (95% CI, −5.7% to 0.7%) and RR was 0.78 (95% CI, 0.60-1.10). In the per-protocol analysis, the RD was −0.1% (95% CI, −3.4% to 3.1%) and RR was 0.99 (95% CI, 0.70-1.40).

**Conclusions and Relevance:**

In this cohort study of uncomplicated gram-negative bacteremia, early transition to oral antibiotics within 4 days of initial blood culture was associated with 90-day all-cause mortality risk comparable to that of continuing IV antibiotic treatment and may be an effective alternative to prolonged IV treatment.

## Key Points

**Question:**

Is risk of 90-day mortality comparable for individuals with uncomplicated gram-negative bacteremia who switch early to oral antibiotics vs continue intravenous (IV) antibiotics?

**Findings:**

In this cohort study using a target trial emulation framework including 914 individuals, similar risks of 90-day all-cause mortality were found in those receiving prolonged IV antibiotics and those switching to oral antibiotics.

**Meaning:**

The results suggest that transition to oral antibiotics within 4 days after initial blood culture may be an effective alternative to prolonged IV antibiotic treatment for uncomplicated gram-negative bacteremia.

## Teaser

This cohort study uses a target trial emulation framework to estimate all-cause mortality outcomes for individuals with uncomplicated gram-negative bacteremia after switching from intravenous to oral antibiotic therapy within 4 days of initial blood culture.

## Introduction

The rising global incidence of gram-negative bacteremia contributes to significant morbidity, mortality, and challenges in clinical management.^1,2^ The optimal treatment duration for gram-negative bacteremia has received increased attention in recent years. While randomized clinical trials (RCTs) have explored this topic, there is a lack of emphasis on the appropriate timing for transitioning from intravenous (IV) to oral antibiotic therapy. Early transition could lead to reduced hospital stays, lower health care costs, and improved patient quality of life.^3,4,5,6,7^ Additionally, switching to oral therapy may help mitigate the risks associated with IV catheters while also facilitating an earlier return to regular activities of daily living.^3,7^

Few studies have directly compared transitioning to oral therapy with continuing IV therapy. A multicenter cohort study compared early oral stepdown therapy and continued IV therapy for gram-negative bacteremia and found comparable outcomes in 30-day all-cause mortality.^5^ Another cohort study focused solely on urinary tract–related gram-negative bacteremia, finding similar treatment failure rates but shorter hospital stays with early oral therapy. Currently, 2 ongoing, open-label RCTs are evaluating the safety and efficacy of early oral antibiotic stepdown in patients with uncomplicated gram-negative bacteremia.^8,9^ The INVEST trial^8^ randomizes patients to either early oral stepdown within 3 days or continuation of IV antibiotic therapy for at least 24 hours after randomization before clinical reassessment. The SOAB trial^9^ randomized patients to either early oral stepdown between days 3 to 5 or continuation of IV treatment for the entire treatment duration. Both trials have excluded patients with complicated gram-negative bacteremia, defined as polymicrobial infection, uncontrolled focus of infection, infection with hard-to-treat gram-negative bacteria, and being immunocompromised or having terminal illness. In both trials, clinical stability, defined as being afebrile and hemodynamically stable, was required for eligibility. The upcoming GOAT trial^10^ will compare oral antibiotic stepdown within 5 days with continuing IV treatment for the duration of therapy but will exclude only patients with polymicrobial infection or immunosuppression or who are actively receiving vasopressors.

Randomized clinical trials are considered the gold standard for evaluating the efficacy and safety of interventions in clinical research. However, RCTs are cost and time intensive. Target trial emulation offers a promising complement to RCTs by leveraging existing data sources to emulate the design and analysis of a hypothetical target trial.^11,12^ This approach aims to estimate the potential effects of interventions by mimicking an idealized RCT using clinical data and evaluating interventions in a timely and cost-effective manner. Our study aimed to estimate the potential effect of an early switch to oral antibiotics compared with continuing IV antibiotics on all-cause mortality among individuals with uncomplicated gram-negative bacteremia by using a target trial framework.

## Methods

### Study Design and Setting

In this cohort study, we specified a hypothetical target trial, drawing inspiration from the INVEST trial protocol,^8^ assigning individuals with uncomplicated gram-negative bacteremia to 1 of 2 treatment arms. One arm was assigned to early transition to oral antibiotics at day 4 after the initial blood culture collection by continuing oral therapy or switching to oral therapy without reinitiating IV antibiotic therapy. The other arm was assigned to continuation of IV antibiotics for a minimum of 24 hours after treatment assignment. Table 1 summarizes the protocol components. The hypothetical target trial was then emulated using observational data. Patients were identified by a blood culture positive for growth of gram-negative bacteria from January 1, 2018, through December 31, 2021. The study was approved by the Danish Data Protection Agency. Informed consent was waived by the Centre for Regional Development because it is not required for register-based studies according to Danish law. The study was reported in accordance with the Strengthening the Reporting of Observational Studies in Epidemiology (STROBE) reporting guideline.^13^

**Table 1.** Specification of the Target Trial and Its Emulation Comparing Early Oral Stepdown Antibiotic Treatment With Continued IV Antibiotic Treatment in Individuals Hospitalized for Gram-Negative Bacteremia

Protocol component	Target trial	Target trial emulation	Data source for emulation
Eligibility criteria	Hospitalized between January 2018 and December 2021	Same as target trial	Electronic health record
	Blood culture with growth of gram-negative bacteremia and evidence of infection^a^	Same as target trial	Department of Clinical Microbiology database and electronic health record
	Age ≥18 y at inclusion	Same as target trial	Electronic health record
	Clinically stable within 4 d of initial blood culture^b^	Same as target trial	Electronic health record
	Susceptibility report available on day 4 after initial blood culture	Same as target trial	Electronic health record
	Initiated appropriate empirical IV antibiotic treatment within 24 h of initial blood culture^c^	Same as target trial	Electronic health record
Exclusion criteria	Immunosuppression^d^	Same as target trial	Electronic health record
	Established uncontrolled focus of infection^e^	Same as target trial	Electronic health record
	Transition to hospice care within 4 d of initial blood culture	Same as target trial	Electronic health record
	Polymicrobial growth in blood culture^f^	Same as target trial	Electronic health record
	Growth of hard-to-treat bacteria in blood culture^g^	Same as target trial	Electronic health record
Treatment strategies	Switch to oral antibiotic treatment within 4 d of initial blood culture and do not reinitiate IV antibiotic treatment throughout the course of antibiotic therapy; the total duration of antibiotic therapy is maintained for 7-14 d	Same as target trial	NA
	Continuation of IV antibiotic treatment beyond 4 d after initial blood culture collection; the total duration of antibiotic therapy is maintained for 7-14 d	Same as target trial	NA
Treatment assignment	Individuals randomized in a 1:1 allocation ratio to a treatment strategy on day 4 after the initial blood culture; individuals informed of their assigned group and aware of their treatment allocation	Individuals classified according to the treatment strategy compatible with their actual antibiotic administration on the index date, defined as day 4 after the initial blood culture; assignment was treated as if randomized within levels of baseline covariates: age, sex, community or nosocomial acquisition, source of infection, pathogen, selected comorbidities, CRP level at time of initial blood culture collection, and Pitt Bacteremia Score	Electronic health record
Follow-up	Started at treatment assignment (day 4) and ended at date of death, loss to follow-up, or 90 d after baseline	Same as target trial	Electronic health record
Primary outcome	90-d All-cause mortality	Same as target trial	Electronic health record
Causal contrasts	Intention-to-treat effect	Observational analogue of intention-to-treat effect	NA
	Per-protocol effect	Observation analogue of per-protocol effect	NA
Analysis plan	Intention-to-treat analysis: Kaplan-Meier survival curves and pooled logistic regression to estimate absolute risk, risk difference, and risk ratio	Inverse probability–weighted Kaplan-Meier survival curves and pooled logistic regression models with flexible time-varying intercept and product terms between treatment and time, adjusting for baseline confounders using inverse probability treatment weights^h^	NA
	Per-protocol analysis: same as the intention-to-treat analysis except individuals were censored when they deviated from the assigned treatment strategy	Same as the target trial; baseline and time-varying factors associated with adherence were adjusted for using the approach as in the intention-to-treat analysis^i^	NA

Abbreviations: CRP, C-reactive protein; IV, intravenous; NA, not applicable.

^a^ Defined as hyperthermia or hypothermia, a localized infection, sepsis, or septic shock.

^b^ Temperature 37.8 °C or lower, systolic blood pressure greater than 90 mm Hg, heart rate less than 100/min, respiratory rate less than 24/min, and peripheral oxygen saturation greater than 90%.

^c^ Acceptable empirical antibiotic treatments were piperacillin-tazobactam, ampicillin in combination with gentamicin, mecillinam, cefuroxime, ceftazidime, cefotaxime, meropenem, ertapenem, and tobramycin.

^d^ Defined as receiving corticosteroid treatment (≥20 mg prednisolone equivalent per day for *>*14 days), being HIV positive, having been treated with chemotherapy for fewer than 28 days, having neutropenia (*<*1000/μL), being an organ transplant recipient, or receiving biological response modifier therapy.

^e^ Undrained abdominal abscess, deep-seated intra-abdominal infection, undrained moderate-severe hydronephrosis, or failure to remove the source of infection within 72 hours of index blood culture.

^f^ Defined as growth of 2 or more different microorganism species in the same blood culture, where contamination was ruled out.

^g^ Defined as bacteremia with growth of nonfermenting gram-negative bacteria (*Acinetobacter*, *Burkholderia*, or *Pseudomonas* species), *Brucella* species, or *Fusobacterium* species.

^h^ Adjusted for age, sex, community or nosocomial acquisition, source of infection, pathogen, selected comorbidities, and CRP level, and Pitt Bacteremia Score at the time of initial blood culture.

^i^ Adjusted for age, community or nosocomial acquisition, source of infection, pathogen, selected comorbidities, CRP level and Pitt Bacteremia Score at blood culture, Pitt Bacteremia Score at index date, discharge prior to index date, and time to discharge during follow-up.

### Data Source

Blood cultures were identified through the Department of Clinical Microbiology at Copenhagen University Hospital–Amager and Hvidovre, which services 4 hospitals in Copenhagen, Denmark. All demographic information and patient characteristics were extracted from electronic health records and securely entered into a REDCap database.^14^ Data on laboratory values were extracted from records of the Department of Clinical Biochemistry.

### Eligibility Criteria

The eligibility criteria included hospitalization, age of 18 years or older, growth of gram-negative bacteria on blood culture, and evidence of infection. Appropriate empirical antibiotic treatment had to be initiated within 24 hours of the first blood culture. Participants had to be clinically stable within 4 days of the initial blood culture (temperature ≤37.8 °C, systolic blood pressure >90 mm Hg, heart rate <100/min, respiratory rate <24/min, and peripheral oxygen saturation >90%), with an available susceptibility report on day 4. Individuals who were immunosuppressed (receiving corticosteroid treatment with ≥20 mg of prednisolone equivalent per day for >14 days, HIV positive, having received chemotherapy for <28 days, neutropenia [<1000 /μL], organ transplant recipient, or receiving biological response modifier therapy), transitioned to hospice care within 4 days of the initial blood culture, had an established uncontrolled focus of infection, or had a blood culture with polymicrobial growth or growth of either *Acinetobacter*, *Burkholderia*, *Pseudomonas*, *Brucella*, or *Fusobacterium* species were excluded. Additionally, individuals who ended antibiotic treatment before or on the index date were excluded.

### Treatment Strategy and Assignment

Early switch to oral antibiotics was defined as transitioning to oral antibiotic therapy within 4 days of blood culture. Prolonged IV treatment was defined as a minimum of 5 days of IV antibiotic treatment.

The index date was set to 4 days after the initial blood culture, mimicking the time of randomization in the target trial. Participants were allocated into 1 of the 2 treatment groups according to the antibiotic treatment strategy with which their data were compatible at the index date.

Appropriate empirical antibiotic treatment was defined as initiation of either piperacillin-tazobactam, ampicillin in combination with gentamicin, mecillinam, cefuroxime, ceftazidime, cefotaxime, meropenem, ertapenem, or tobramycin. Choice of IV and oral antibiotic agent after availability of the susceptibility report was at the discretion of the treating physician but had to comply with the susceptibility result. Antibiotic treatment had to be maintained for 7 to 14 days.

### Follow-Up and Outcomes

Follow-up started at randomization and ended on day 90 or at the time of death, whichever occurred first. The primary outcome was 90-day all-cause mortality.

### Statistical Analysis

Participant characteristics are presented as medians and IQRs for continuous variables. For categorical variables, characteristics are reported as frequencies and percentages.

For the intention-to-treat analysis, we estimated the 90-day risk, risk difference (RD), and risk ratio (RR) for all-cause mortality using a pooled logistic regression model with a flexible time-varying intercept (estimated as linear and quadratic terms of time since the index date) and product terms between treatment and time.^15^ We adjusted for baseline confounders using inverse probability of treatment weighting (IPTW), which was calculated using age, sex, location of infection acquisition (ie, community, nosocomial), source of infection, pathogen, selected comorbidities (liver disease, kidney disease, cardiovascular disease, diabetes, and solid cancer), and C-reactive protein (CRP) level and Pitt Bacteremia Score (PBS) at the time of blood culture. Covariates were chosen a priori as informed by literature and clinical knowledge and assessed in directed acyclic graphs (eFigure 1 in Supplement 1). Further details on covariates are presented in eTable 1 in Supplement 1.

For the per-protocol analysis, participants were censored if and when they deviated from their assigned treatment strategy; participants in the early-switch group were censored if they reinitiated IV antibiotics. Participants in both groups were censored if the total antibiotic treatment duration was less than 7 or more than 14 days. The 90-day risk, RD, and RR of all-cause mortality were calculated using a pooled logistic regression. To adjust for the potential selection bias introduced by censoring for nonadherence at each day after the index date, time-varying inverse probability weights were used to adjust for baseline and time-varying factors associated with treatment adherence (age, community vs nosocomial acquisition, source of infection, pathogen, selected comorbidities, CRP and PBS at time of blood culture, PBS at index date, discharge prior to index date, and time to discharge during follow-up). An overview of the study design, treatment allocation, and censoring process is shown in eFigure 2 in Supplement 1.

For both the intention-to-treat analysis and the per-protocol analysis, weights were truncated at their 99th percentile to prevent outliers with extreme weights from influencing the effect estimates and to avoid possible model misspecification, but the use of nontruncated weights yielded similar estimates. Nonparametric bootstrapping with 500 samples was used to calculate 95% CIs.

Standardized mean differences in confounder variables between groups were calculated before and after IPTW. Inverse probability–weighted Kaplan-Meier survival curves were generated. Statistical analysis was performed using R, version 4.2.2 (R Project for Statistical Computing).^16^

#### Sensitivity and Subgroup Analyses

To assess the robustness of the model, multiple sensitivity analyses were conducted to evaluate the effect of modifying the preplanned analysis specified in the target trial protocol. In separate analyses, (1) IV antibiotic initiation was varied to 12 hours or less, (2) clinical stability was set at 3 days after blood culture, (3) stratification was performed by infection source (urinary tract infection) and age (<75 and ≥75 years), (4) the Charlson Comorbidity Index (CCI)^17^ was used in place of selected comorbidities, (5) the modified Sequential Organ Failure Assessment score was used instead of the PBS, (6) analysis was restricted to individuals with available plasma CRP data at the index date, (7) immunosuppressed individuals were included, and (8) post hoc analysis on 30-day all-cause mortality was performed.

#### Missing Data

We had complete data for all variables included in the main analysis. Plasma CRP data at the index date were missing for some individuals. Assuming individuals with a better response to antibiotic treatment lacked follow-up CRP measurements, index-date CRP levels were included in a complete case-sensitivity analysis.

## Results

### Baseline Characteristics

**Figure 1.**
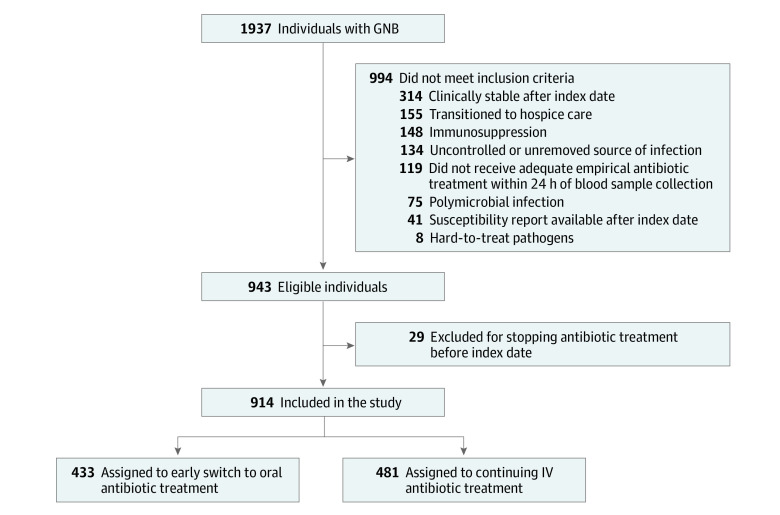
Selection of Individuals With Gram-Negative Bacteremia (GNB) for the Target Trial Emulation IV indicates intravenous.

Of 914 eligible individuals (median age, 74.5 years [IQR, 63.3-83.2 years]; 402 [44.0%] female; 512 [56.0%] male), 433 (47.4%) switched early to oral antibiotic treatment while 481 (52.6%) received prolonged IV treatment (Figure 1). Table 2 details baseline characteristics in the emulated trial. Compared with individuals receiving prolonged IV treatment, individuals switching early to oral antibiotic treatment were younger (median age, 73.0 years [IQR, 60.5-81.6 years] vs 76.0 years [IQR, 66.8-84.6 years]), had fewer comorbidities (median CCI score, 4 [IQR, 2-5] vs 5 [IQR, 3-6]), were more likely to have community-acquired gram-negative bacteremia (387 of 433 [89.4%] vs 389 of 481 [80.9%]) and the urinary tract as the source of the infection (360 of 433 [83.1%] vs 339 of 481 [70.5%]), and had lower median plasma CRP values at the time of the initial blood culture (11.0 mg/dL [IQR, 5.0-21.0 mg/dL] vs 14.0 mg/dL [IQR, 7.2-25.0 mg/dL]) (to convert to mg/L, multiply by 10). Oral β-lactams were prescribed to most of the cohort (577 [63.1%]), oral ciprofloxacin was prescribed to 152 (16.6%), and less than 1% (4) received sulfamethoxazole-trimethoprim.

**Table 2.** Baseline Characteristics of Eligible Individuals With GNB in the Target Trial Emulation

Characteristic	Patients^a^			SMD	
	Total (N = 914)	Prolonged IV antibiotics (n = 481)	Early oral antibiotic switch (n = 433)	Before IPTW	After IPTW
Age, median (IQR), y	74.5 (63.3-83.2)	76.0 (66.8-84.6)	73.0 (60.5-81.6)	0.28	0.003
Sex					
Female	402 (44.0)	220 (45.7)	182 (42.0)	0.08	0.007
Male	512 (56.0)	261 (54.3)	251 (58.0)		
GNB acquisition					
Community	776 (84.9)	389 (80.9)	387 (89.4)	0.24	0.025
Hospital-associated					
Hospitalization and discharge within 14 d of current admission	80 (8.8)	52 (10.8)	28 (6.5)		
Nosocomial^b^	58 (6.3)	40 (8.3)	18 (4.1)		
Source of bacteremia					
Overall	NA	NA	NA	0.31	0.025
Urinary tract infection	699 (76.5)	339 (70.5)	360 (83.1)	0.30	
Gatrointestinal infection	156 (17.1)	104 (21.6)	52 (12.0)	0.26	
Gastrointestinal surgery	16 (1.8)	10 (2.1)	6 (1.4)	0.05	
Pneumonia	17 (1.9)	10 (2.1)	7 (1.6)	0.03	
Other	26 (2.8)	18 (3.7)	8 (1.8)	0.12	
ICU during hospitalization	10 (1.1)	8 (1.7)	2 (0.5)	0.12	NA
mSOFA, median (IQR)	1 (0-2)	2 (0-3)	1 (0-2)	0.42	NA
CCI score, median (IQR)	4 (3-6)	5 (3-6)	4 (2-5)	0.30	0.004
Liver disease	36 (3.9)	24 (5.0)	12 (2.8)	0.12	NA
Kidney disease	61 (6.7)	36 (7.5)	25 (5.8)	0.07	NA
Cardiovascular disease	293 (32.1)	171 (35.6)	122 (28.2)	0.16	NA
Diabetes	236 (25.8)	141 (29.3)	95 (21.9)	0.17	NA
Solid cancer	124 (13.6)	72 (15.0)	52 (12.0)	0.09	NA
CRP level, median (IQR), mg/L					
Baseline	13.0 (6.0-23.0)	14.0 (7.2-25.0)	11.0 (5.0-21.0)	0.24	0.009
Index date	7.2 (4.5- 10.0)	8.6 (5.6-13.0)	5.6 (3.8-8.3)	0.71	NA
Missing	106 (11.6)	32 (6.7)	74 (17.1)	NA	NA
Pitt Bacteremia Score at baseline, median (IQR)	0 (0-1)	0 (0-1)	0 (0-1)	0.13	0.001
Pathogen					
Overall	NA	NA	NA	0.21	0.004
* Escherichia coli*	693 (75.8)	349 (72.6)	344 (79.4)	0.16	
*Klebsiella* species	114 (12.5)	73 (15.2)	41 (9.5)	0.17	
*Enterobacter* species	28 (3.1)	16 (3.3)	12 (2.8)	0.03	
*Proteus* species	30 (3.3)	20 (4.2)	10 (2.3)	0.11	
Other	49 (5.4)	23 (4.8)	26 (6.0)	0.05	
Antimicrobial resistance					
Extended-spectrum β-lactamase producing	5 (0.5)	4 (0.8)	1 (0.2)	NA	NA
Ciprofloxacin	100 (10.9)	64 (13.3)	36 (8.3)	NA	NA
Sulfamethoxazole-trimethoprim	157 (17.3)	94 (19.5)	63 (14.5)	NA	NA

Abbreviations: CCI, Charlson Comorbidity Index; CRP, C-reactive protein; GNB, gram-negative bacteremia; ICU, intensive care unit; IPTW, inverse probability of treatment weighting; IV, intravenous; mSOFA, modified sequential organ failure assessment; NA, not applicable; SMD, standardized mean difference.

SI conversion factor: To convert CRP to mg/L, multiply by 10.

^a^ Data are presented as number (percentage) of patients unless otherwise indicated.

^b^ Occurred 48 hours or longer after hospital admission.

The distribution of the applied weights was satisfactory, with a mean close to 1.0 and a maximum weight of 2.5. Confounder variables demonstrated a well-balanced distribution between groups after weighting, with no standardized mean differences exceeding 0.025.

### Primary Outcome

Ninety-nine individuals (10.8%) died during follow-up. The proportion of individuals who died was higher in the group receiving prolonged IV treatment (69 of 481 [14.3%] vs 3 of 433 [6.9%]). In the intention-to-treat analysis, 90-day all-cause mortality risk was 9.1% (95% CI, 6.7%-11.6%) for the early switch group and 11.7% (95% CI, 9.6%-13.8%) for the group receiving prolonged IV treatment, corresponding to an RD of −2.5% (95% CI, −5.7 to 0.7) and an RR of 0.78 (95% CI, 0.60-1.10) (Table 3).

**Table 3.** Inverse Probability–Weighted 90-Day Risk of All-Cause Mortality Among Individuals With Gram-Negative Bacteremia Continuing IV Antibiotic Therapy vs Early Switch to Oral Antibiotic Therapy

Analysis	90-d Risk of all-cause mortality, % (95% CI)		90-d Risk difference (95% CI)	90-d Risk ratio (95% CI)
	Early oral switch	Continuing IV treatment		
Intention-to-treat	9.1 (6.7-11.6)	11.7 (9.6-13.8)	−2.5 (−5.7 to 0.7)	0.78 (0.60 to 1.10)
Per-protocol	9.6 (6.7-12.4)	9.7 (7.6-11.8)	−0.1 (−3.4 to 3.1)	0.99 (0.70 to 1.40)

Abbreviation: IV, intravenous.

**Figure 2.**
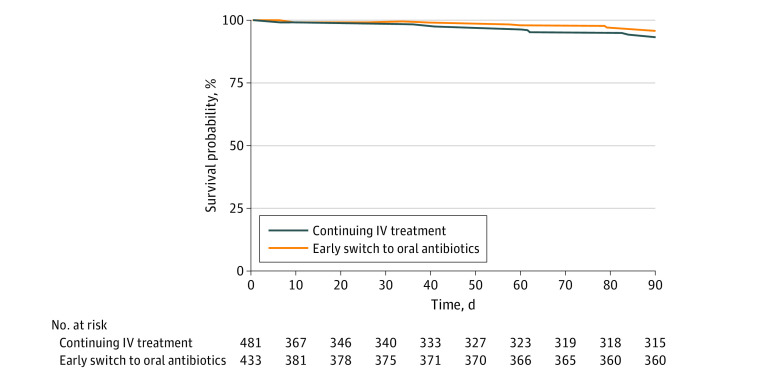
Weighted Survival Curves for Individuals Who Continued or Switched to Early Oral Antibiotics IV indicates intravenous.

In the per-protocol analysis, 747 individuals (81.7% of 914 in the intention-to-treat population) adhered to the study protocol. Adherence was 89.4% (n = 387) in the early-switch group and 74.8% (n = 360) in the group receiving prolonged IV antibiotics. The primary reason for nonadherence was a total treatment duration shorter than 7 days or longer than 14 days. No individuals in the early-switch group reinitiated IV treatment. No individuals were lost-to-follow-up. The 90-day risk of all-cause mortality was 9.6% (95% CI, 6.7%-12.4%) for the early-switch group and 9.7% (95% CI, 7.6%-11.8%) for the group continuing IV antibiotic treatment, corresponding to an RD of −0.1% (95% CI, −3.4% to 3.1%) and an RR of 0.99 (95% CI, 0.70-1.40). Post hoc power analysis demonstrated 93.2% power to detect differences in 90-day mortality. Weighted survival curves are shown in Figure 2.

### Sensitivity Analyses and Subgroup Analyses

Sensitivity analyses are presented in eTable 2 in Supplement 1. In a 30-day follow-up period, 45 individuals died (13 of 433 [3.0%] in the early-switch group and 32 of 481 [6.7%] in the prolonged IV group). The per-protocol adjusted RD was −1.2% (95% CI, −4.8% to 2.4%).

Plasma CRP data at the index date were missing for 106 individuals (11.6%). The sensitivity analyses including CRP levels at the index date and CCI scores instead of selected comorbidities yielded estimates comparable to those of the main analysis.

Stricter eligibility criteria reduced the sample size. Among individuals achieving clinical stability within 3 days, the per-protocol adjusted RD was −1.4% (95% CI, −5.2% to 2.5%). When antibiotic treatment was initiated within 12 hours of blood culture, the per-protocol adjusted RD was −1.2% (95% CI, −4.5% to 2.2%).

The inclusion of immunosuppressed individuals (n = 77) expanded the sample size to 991. Thirty-five individuals with immunosuppression (45.5%) received early oral stepdown treatment, while 42 (54.5%) received prolonged IV treatment. The results of the intention-to-treat and per-protocol analyses were consistent with those of the main analysis.

Subgroup analyses are presented in eTable 3 in Supplement 1. For individuals with a urinary tract infection as the source of infection and for those younger than 75 years, the adjusted 90-day mortality risk was lower in both the intention-to-treat and the per-protocol analyses compared with the main analysis. For individuals older than 75 years, the risk in the intention-to-treat analysis was higher compared with the main analysis, while the per-protocol analysis yielded results similar to those of the main analysis.

## Discussion

This study used a target trial framework to estimate the potential effectiveness of early oral stepdown therapy in individuals with uncomplicated gram-negative bacteremia. Overall, we found comparable rates of 90-day all-cause mortality between clinically stable individuals transitioning early to oral antibiotics compared with individuals receiving prolonged IV antibiotic treatment.

These results align with those of previous studies. A retrospective study by Rieger et al^6^ found no difference in treatment failure risk in individuals who transitioned to oral antibiotic therapy for gram-negative bacteremia with urinary tract infection as the source of infection compared with individuals receiving IV treatment. Similarly, a propensity score–matched cohort study investigating early oral antibiotic switch within 5 days of treatment initiation for gram-negative bacteremia found no between-group difference in 30-day mortality.^5^ The study included patients with immunosuppression, a group initially excluded from our primary analysis. However, when this group was included in our analysis, the difference in 90-day all-cause mortality remained comparable between the 2 treatment groups, with an even distribution of individuals with immunosuppression in both groups.

As anticipated, individuals in our study who received prolonged IV antibiotics were older, had more severe progression of bacteremia, and had a higher burden of comorbidities compared with individuals switching early to oral treatment. Although IPTW adjusted for these differences at baseline, we hypothesized that receiving antibiotics beyond the defined total treatment duration might indicate a more complex disease progression. The observed larger RD in the intention-to-treat analysis compared with the per-protocol analysis may be indicative of these underlying factors. The per-protocol analysis adjusted for both baseline and time-varying confounders, in which individuals deviating from the assigned treatment strategy were censored, thus leading to a more accurate estimation.

In terms of 30-day all-cause mortality, the intention-to-treat and per-protocol analyses yielded comparable results, indicating an RD of –1.2%. However, these results should be interpreted considering the low 30-day event rate.

Not surprisingly, we found a higher mortality risk in the intention-to-treat analysis in the subgroup including patients older than 75 years, as the older subpopulation was more likely to have severe disease progression and a higher burden of comorbidities. However, the per-protocol analysis yielded the same absolute risk as the main analysis, suggesting no difference in risk between the 2 treatment groups despite the age difference. In all analyses, the upper limits of the 95% CIs were below the 6% noninferiority margin used in the INVEST trial.^8^

Two studies^18,19^ have explored the current treatment pattern among patients with uncomplicated gram-negative bacteremia and found that just one-fifth were transitioned to oral antibiotics within 5 days, thus highlighting the need for further studies and information to establish the optimal timing of and criteria for oral stepdown treatment. Prior studies reported success for patients with uncomplicated bacteremia switching to oral treatment within 3 to 5 days.^20,21^ Current guidelines^22^ recommend a total treatment duration of 7 days for uncomplicated gram-negative bacteremia, and a recent study suggested that 5 days of treatment for patients showing clinical response guided by CRP values may be sufficient in the treatment of gram-negative bacteremia.^23^ As the eligibility criteria in our cohort required patients to achieve clinical stability before day 5, a cutoff prior to day 5 may seem more reasonable.

### Strengths and Limitations

This study has several strengths. First, the target trial emulation framework allowed us to include retrospective observational data to address a clinical question for which there is currently no RCT evidence, to our knowledge. The application of the principles of an RCT to an observational study minimizes bias while including individuals that would not necessarily be included in an RCT, thereby broadening the clinical relevance of the results. Second, access to highly detailed data allowed us to adjust for relevant baseline and time-varying variables, leading to a more accurate estimation of the treatment effect. Third, the multicenter design allowed us to account for variations in treatment patterns. Fourth, due to records of the entire Danish population in the Civil Registration System being linked to electronic health records, we could achieve complete follow-up, avoiding observer bias.

Our study has certain limitations. As it was an observational study based on information from electronic health records, important confounders may have been incompletely recorded or absent, and it is possible that we were unable to consider all variables affecting the decision to switch patients to oral stepdown treatment. For example, CRP values at the index date were not obtained for the whole population and thus were included only in a sensitivity analysis, and information on adherence to treatment after discharge was not recorded. Furthermore, it is important to highlight that the incidence of multitherapy-resistant infections in this study was minimal, which could potentially affect the applicability of our findings. Lastly, as our study cohort was characterized by achieving clinical stability by day 4 at the latest, a substantial number of individuals with severe or complicated gram-negative bacteremia were omitted from the analysis. Consequently, the results cannot be extended to encompass all instances of gram-negative bacteremia; rather, they are applicable to uncomplicated cases with clinical response within 4 days.

## Conclusions

This cohort study using a target trial emulation framework investigating early stepdown from IV to oral antibiotics for patients with uncomplicated gram-negative bacteremia estimated comparable 90-day all-cause mortality risk between treatment groups. Results were consistent across age groups and among individuals with immunosuppression. These findings suggest that the mortality associated with early antibiotic stepdown treatment is comparable to that associated with receiving prolonged IV antibiotic treatment for individuals with uncomplicated gram-negative bacteremia.

## Notes

Supplement 1. **eFigure 1.** Direct Acyclic Graph Depicting the Hypothesized Association of Early Switch to Oral Antibiotics With All-Cause Mortality **eTable 1.** Covariate Definitions in the Target Trial **eFigure 2.** Hypothetical Treatment Allocation and Censoring Process in Target Trial Emulation **eTable 2.** Sensitivity Analyses on 90-Day Mortality of Individuals With Gram-Negative Bacteremia Receiving Prolonged Intravenous Compared With Early Switch to Oral Antibiotic Therapy **eTable 3.** Subgroup Analyses on 90-Day Mortality of Individuals With Gram-Negative Bacteremia Continuing Intravenous Compared With Early Switch to Oral Antibiotic Therapy

Supplement 2.
Data Sharing Statement
