# Supplementary material for: Early Switch From Intravenous to Oral Antibiotics for Patients With Uncomplicated Gram-Negative Bacteremia
Source: JAMA Netw Open. 2024 Jan 23;7(1):e2352314. doi: 10.1001/jamanetworkopen.2023.52314 (PMC10807296; doi:10.1001/jamanetworkopen.2023.52314)
Supplement: Supplement 1. — eFigure 1. Direct Acyclic Graph Depicting the Hypothesized Association of Early Switch to Oral Antibiotics With All-Cause Mortality eTable 1. Covariate Definitions in the Target Trial eFigure 2. Hypothetical Treatment Allocation and Censoring Process in Target Trial Emulation eTable 2. Sensitivity Analyses on 90-Day Mortality of Individuals With Gram-Negative Bacteremia Receiving Prolonged Intravenous Compared With Early Switch to Oral Antibiotic Therapy eTable 3. Subgroup Analyses on 90-Day Mortality of Individuals With Gram-Negative Bacteremia Continuing Intravenous Compared With Early Switch to Oral Antibiotic Therapy [file jamanetwopen-e2352314-s001.pdf]

Supplementary Online Content

Tingsgård S, Bastrup Israelsen S, Jørgensen HL, Østergaard C, Benfield T. Early switch from intravenous to oral antibiotics for patients with uncomplicated gram-negative bacteremia. *JAMA Netw Open*. 2024;7(1):e2352314. doi:10.1001/jamanetworkopen.2023.52314

eFigure 1. Direct Acyclic Graph Depicting the Hypothesized Association of Early Switch to Oral Antibiotics With All-Cause Mortality

eTable 1. Covariate Definitions in the Target Trial

eFigure 2. Hypothetical Treatment Allocation and Censoring Process in Target Trial Emulation

eTable 2. Sensitivity Analyses on 90-Day Mortality of Individuals With Gram-Negative Bacteremia Receiving Prolonged Intravenous Compared With Early Switch to Oral Antibiotic Therapy

eTable 3. Subgroup Analyses on 90-Day Mortality of Individuals With Gram-Negative Bacteremia Continuing Intravenous Compared With Early Switch to Oral Antibiotic Therapy

This supplementary material has been provided by the authors to give readers additional information about their work.

© 2024 Tingsgård S et al. *JAMA Network Open*.

eFigure 1. Direct Acyclic Graph depicting the hypothesized association of early oral switch (exposure) and all-cause mortality (outcome)

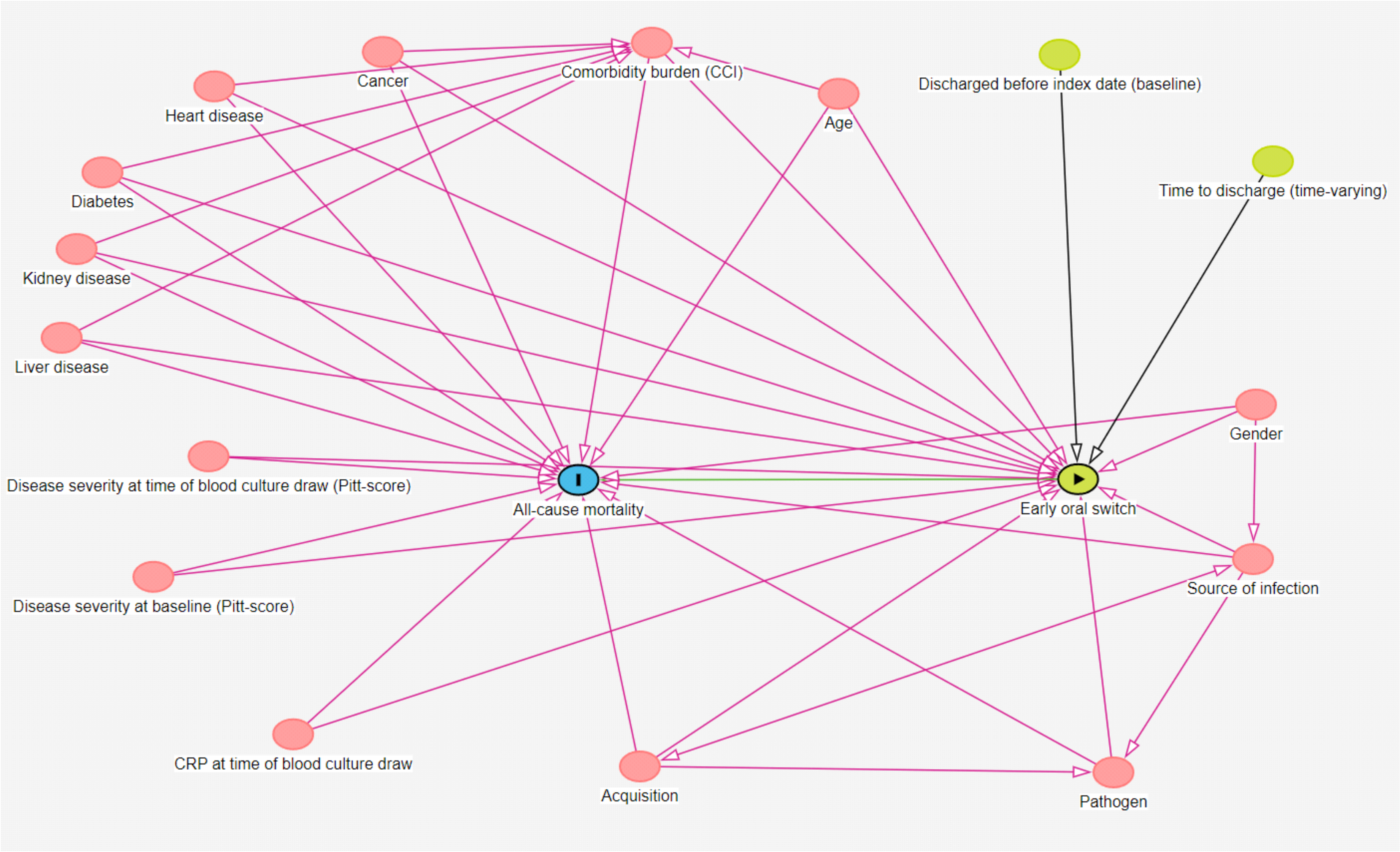

CRP, C-reactive protein; CCI, Charlson comorbidity index

© 2024 Tingsgård S et al. *JAMA Network Open*.

eTable 1. Covariate definitions in the target trial

Covariate	Data source	Definition (if further detail required)	Indicator	Categories
Age	Medical record		Linear, quadratic	NA
Gender	Medical record		Dichotomous	Male/female
Pitt Bacteremia score	Medical record	Score from 0-12 points calculated from following:<br>*Temperature (°C):*<br>• 36.1-38.9 = 0 point<br>• 35.1-36.0 / 39.0-39.9 = 1 point<br>• ≥40 = 2 points<br>*Hypotension:*<br>• Fall in sBP >30 mmHg / fall in dBP >20 mmHg / need for vasopressors / sBP <90 mmHg = 2 points<br>• No hypotension as defined above = 0 point<br>*Need for mechanical ventilation:*<br>• Yes = 2 points<br>• No = 0 point<br>*Cardiac arrest*<br>• Yes = 4 points<br>• No = 0 point<br>*Mental state*<br>• Comatose = 4 points<br>• Stuporous = 2 points<br>• Disoriented = 1 point<br>• Alert = 0 point	Linear	NA
CRP	Medical record		Linear	NA

© 2024 Tingsgård S et al. *JAMA Network Open*.

Covariate	Data source	Definition (if further detail required)	Indicator	Categories
Pathogen	Medical record	1: Escherichia coli<br>2: Klebsiella spp.<br>3: Enterobacter spp.<br>4: Proteus spp.<br>5: Other	5 categories	1, 2, 3, 4, 5
Hard-to-treat pathogen	Medical record	Includes *Acinetobacter* spp., *Burkholderia* spp., *Pseudomonas* spp., *Brucella* spp., and *Fusobacterium* spp.	Dichotomous	Yes/no
Acquisition	Medical record		Dichotomous	Community-acquired/hospital-associated
Source	Medical record	1: Urinary tract infection<br>2: Gastrointestinal infection<br>3: Gastrointestinal surgery<br>4: Pneumonia<br>5: Other	5 categories	1, 2, 3, 4, 5
Liver disease	Medical record	All severities	Dichotomous	Yes/no
Renal disease	Medical record	All severities	Dichotomous	Yes/no
Solid cancer	Medical record		Dichotomous	Yes/no
Cardiovascular disease	Medical record	Hypertension not included	Dichotomous	Yes/no
Diabetes	Medical record		Dichotomous	Yes/no
Immunosuppression	Medical record	Defined as receiving corticosteroid treatment (≥20 mg prednisolone-equivalent/day > 14 days), being HIV-positive, having been treated with chemotherapy <28 days, having neutropenia (< 1000/μL), being an organ transplant recipient, or	Dichotomous	Yes/no

© 2024 Tingsgård S et al. *JAMA Network Open*.

Covariate	Data source	Definition (if further detail required)	Indicator	Categories
		receiving biological response modifier therapy		
Discharged before index date	Medical record		Dichotomous	Yes/no
Time to discharge during follow-up	Medical record	Calculated in days<br>Time-varying covariate	Linear	NA

Abbreviation: sBP, systolic blood pressure; dBP, diastolic blood pressure; HIV, human immunodeficiency virus; CRP, C-reactive protein; spp, species; NA: not applicable

© 2024 Tingsgård S et al. *JAMA Network Open*.

eFigure 2. Hypothetical treatment allocation and censoring process in target trial emulation

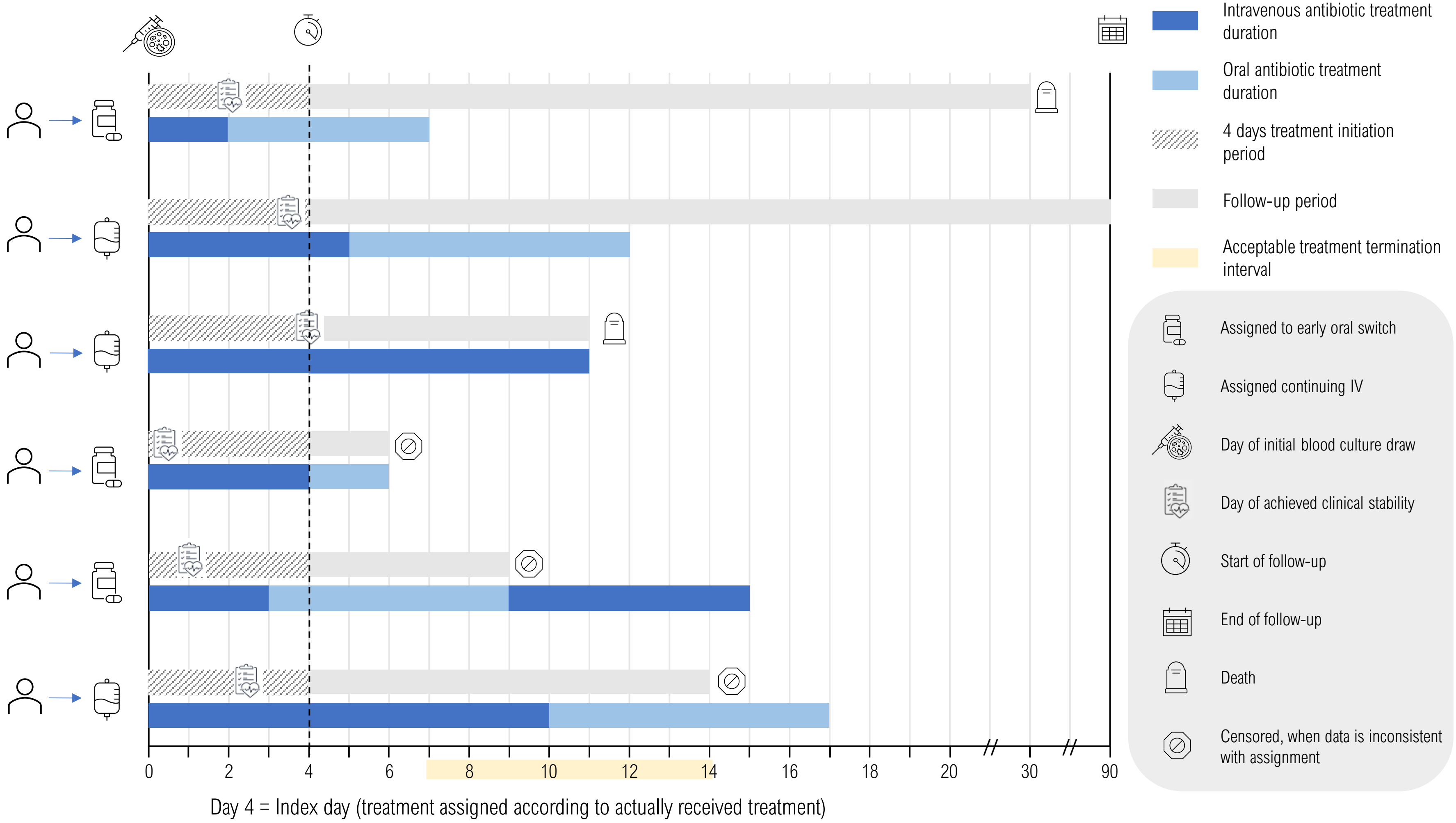

© 2024 Tingsgård S et al. *JAMA Network Open*.

eTable 2. Sensitivity analyses on 90-day mortality of individuals with Gram-negative bacteremia receiving prolonged intravenous compared with early switch to oral antibiotic therapy

	**90-day risk of all-cause mortality (%, 95% CI)**		**90-day risk difference (95% CI)**	**90-day risk ratio (95% CI)**
	**Early oral switch**	**Prolonged IV**		
**30-day all-cause mortality**				
Estimated intention-to-treat	4.2 (2.4, 6.0)	5.5 (3.9, 7.0)	-1.2 (-3.4, 1.0)	0.77 (0.45,1.33)
Estimated per-protocol	4.7 (1.6, 7.9)	5.9 (3.5, 8.3)	-1.2 (-4.8, 2.4)	0.79 (0.36, 1.74)
**CCI-score instead of selected comorbidities (n = 914)**				
Estimated intention-to-treat	9.0 (6.6, 11.4)	11.6 (9.7, 13.6)	-2.7 (-5.7, 0.4)	0.77 (0.60, 1.07)
Estimated per-protocol	9.4 (6.7, 12.1)	9.8 (7.7, 12.0)	-0.4 (-3.8, 2.8)	0.95 (0.70, 1.40)
**CRP at index date included in the model (n = 808)**				
Estimated intention-to-treat	9.8 (7.5,12.1)	12.2 (10.0,14.5)	-2.4 (-5.5,0.7)	0.80 (0.59,1.09)
Estimated per-protocol	10.1 (7.5,12.6)	10.2 (7.8,12.6)	-0.1 (-3.2,2.9)	0.99 (0.72,1.36)

© 2024 Tingsgård S et al. *JAMA Network Open*.

**Including individuals with immunosuppression**				
**(n = 991)**				
Estimated intention-to-treat	9.6 (7.3,12.0)	12.1 (10.1,14.1)	-2.5 (-5.4,0.4)	0.79 (0.59,1.07)
Estimated per-protocol	9.4 (6.7,12.0)	9.7 (7.3,12.1)	-0.3 (-4.1,3.4)	0.97 (0.63,1.48)
**Achieved clinical stability ≤3 days**				
**(n = 863)**				
Estimated intention-to-treat	7.9 (5.8, 9.9)	11.6 (9.4, 13.8)	-3.7 (-6.9, -0.6)	0.68 (0.5, 1.0)
Estimated per-protocol	8.2 (5.6, 10.8)	9.6 (7.0, 12.1)	-1.4 (-5.2, 2.5)	0.86 (0.5, 1.4)
**Initiation of empirical antibiotic treatment ≤12 hours**				
**(n = 763)**				
Estimated intention-to-treat	8.6 (5.8, 11.4)	11.5 (9.5, 13.4)	-2.9 (-6.3, 0.6)	0.75 (0.5, 1.1)
Estimated per-protocol	8.5 (5.8, 11.3)	9.7 (7.7, 11.7)	-1.2 (-4.5, 2.2)	0.88 (0.6, 1.3)

Abbreviation: CI, confidence interval; IV, intravenous; CCI, Charlson Comorbidity Index; CRP, C-reactive Protein

© 2024 Tingsgård S et al. *JAMA Network Open*.

eTable 3. Subgroup analyses on 90-day mortality of individuals with Gram-negative bacteremia continuing intravenous compared with early switch to oral antibiotic therapy

	90-day risk of all-cause mortality (%, 95% CI)		90-day risk difference (95% CI)	90-day risk ratio (95% CI)
	Early oral switch	Continuing IV		
**Urinary tract infection as source of bacteremia (n = 699)**				
Estimated intention-to-treat	7.2 (4.8, 9.6)	8.6 (6.5, 10.7)	-1.4 (-4.7, 1.9)	0.84 (0.5, 1.4)
Estimated per-protocol	7.0 (4.8, 9.2)	8.1 (5.6, 10.6)	-1.1 (-4.2, 2.0)	0.87 (0.6, 1.4)
**Age <75 years (n = 466)**				
Estimated intention-to-treat	4.4 (2.3, 6.5)	6.2 (4.0, 8.4)	-1.8 (-5.0, 1.3)	0.70 (0.3, 1.4)
Estimated per-protocol	4.0 (1.7, 6.2)	4.4 (0.7, 8.1)	-0.4 (-5.6, 4.7)	0.90 (0.3, 3.1)
**Age ≥75 years (n = 448)**				
Estimated intention-to-treat	13.3 (9.1, 17.6)	17.8 (14.3, 21.2)	-4.4 (-10.2, 1.4)	0.75 (0.5, 1.2)
Estimated per-protocol	9.4 (6.7, 12.1)	9.8 (7.7, 12.0)	-0.4 (-3.8, 2.8)	0.95 (0.7, 1.4)

Abbreviation: CI, confidence interval; IV, intravenous

© 2024 Tingsgård S et al. *JAMA Network Open*.
