# Supplementary material for: Early Switch From Intravenous to Oral Antibiotics for Patients With Uncomplicated Gram-Negative Bacteremia
Source: JAMA Netw Open. 2024 Jan 23;7(1):e2352314. doi: 10.1001/jamanetworkopen.2023.52314 (PMC10807296; doi:10.1001/jamanetworkopen.2023.52314)
Supplement: Supplement 2. — Data Sharing Statement [file jamanetwopen-e2352314-s002.pdf]

Data Sharing Statement

Tingsgård. Early Switch From Intravenous to Oral Antibiotics for Patients With Uncomplicated Gram-Negative Bacteremia. *JAMA Netw Open*. Published January 23, 2024. doi:10.1001/jamanetworkopen.2023.52314

Data
Data available: No

Additional Information
Explanation for why data not available: According to the Danish Data Protection Agency and the General Data Protection Regulation (GDPR), the sharing of sensitive data is prohibited. However, anonymous data may be shared upon a reasonable request made to the corresponding author.
